# Analysis of Prognostic Factors of Rectal Cancer and Construction of a Prognostic Prediction Model Based on Bayesian Network

**DOI:** 10.3389/fpubh.2022.842970

**Published:** 2022-06-17

**Authors:** Ruikai Li, Chi Zhang, Kunli Du, Hanjun Dan, Ruxin Ding, Zhiqiang Cai, Lili Duan, Zhenyu Xie, Gaozan Zheng, Hongze Wu, Guangming Ren, Xinyu Dou, Fan Feng, Jianyong Zheng

**Affiliations:** ^1^Department of Gastrointestinal Surgery, Xijing Hospital, Fourth Military Medical University, Xi'an, China; ^2^Department of Industrial Engineering, School of Mechantronics, Northwestern Polytechnical University, Xi'an, China; ^3^Department of Cell Biology and Genetics, Medical College of Yan'an University, Yan'an, China; ^4^Graduate Work Department, Xi'an Medical University, Xi'an, China

**Keywords:** Bayesian network, clinicopathological factor, prediction model, prognosis, rectal cancer

## Abstract

**Background:**

The existing prognostic models of rectal cancer after radical resection ignored the relationships among prognostic factors and their mutual effects on prognosis. Thus, a new modeling method is required to remedy this defect. The present study aimed to construct a new prognostic prediction model based on the Bayesian network (BN), a machine learning tool for data mining, clinical decision-making, and prognostic prediction.

**Methods:**

From January 2015 to December 2017, the clinical data of 705 patients with rectal cancer who underwent radical resection were analyzed. The entire cohort was divided into training and testing datasets. A new prognostic prediction model based on BN was constructed and compared with a nomogram.

**Results:**

A univariate analysis showed that age, Carcinoembryonic antigen (CEA), Carbohydrate antigen19-9 (CA19-9), Carbohydrate antigen 125 (CA125), preoperative chemotherapy, macropathology type, tumor size, differentiation status, T stage, N stage, vascular invasion, KRAS mutation, and postoperative chemotherapy were associated with overall survival (OS) of the training dataset. Based on the above-mentioned variables, a 3-year OS prognostic prediction BN model of the training dataset was constructed using the Tree Augmented Naïve Bayes method. In addition, age, CEA, CA19-9, CA125, differentiation status, T stage, N stage, KRAS mutation, and postoperative chemotherapy were identified as independent prognostic factors of the training dataset through multivariate Cox regression and were used to construct a nomogram. Then, based on the testing dataset, the two models were evaluated using the receiver operating characteristic (ROC) curve. The results showed that the area under the curve (AUC) of ROC of the BN model and nomogram was 80.11 and 74.23%, respectively.

**Conclusion:**

The present study established a BN model for prognostic prediction of rectal cancer for the first time, which was demonstrated to be more accurate than a nomogram.

## Introduction

Rectal cancer is the eighth most common malignancy worldwide with a high mortality rate, resulting in about 340,000 deaths every year (1), and has become one of the major public health problems threatening human health. Despite the involvement of chemoradiotherapy and immunotherapy, the prognosis of rectal cancer has not improved significantly, and radical resection is still the primary treatment for rectal cancer at present (2). Prediction of the prognosis of rectal cancer is very important to the management of patients. The greatest significance of a more accurate prediction of survival is that it can effectively avoid excessive treatment and waste of medical resources, and at the same time provide a scientific basis for medical staff and patients to make medical decisions, such as whether to accept postoperative chemotherapy. In addition, it helps patients plan for the rest of their life and makes the best use of time to achieve some aspirations and make life more fulfilling. A series of methods based on clinical data have been applied to the analysis of prognostic factors for patients with rectal cancer. However, these studies only evaluate the separate impacts of individual parameters, such as age, surgical type, and body mass index (BMI) (3–5). In recent years, some prognostic studies based on multivariate survival analysis have become popular. Fan et al. screened out 8 independent prognostic clinicopathological factors (age, sex, preoperative CEA, perineural invasion, tumor deposits, tumor grade, T stage, and N stage) for non-metastatic rectal cancer, and constructed a prognostic prediction nomogram with the concordance index (C-index) of 0.71 (6). Liu et al. screened out 5 independent prognostic pathological factors (yp T stage, yp N stage, tumor location, differentiation status, and postoperative chemotherapy) and constructed a nomogram with a C-index of 0.72 through multivariate analysis of the prognosis of patients with rectal cancer who received neoadjuvant therapy (7). Nevertheless, these studies ignored the cause-and-effect relationships between these prognostic factors. The interaction between these factors and their mutual influences is not yet clear, so an effective modeling method is needed to analyze and represent the relationships among these factors.

A Bayesian network (BN) is a directed acyclic graph used to represent the causal relationship between random events and is a tool to apply probability and statistics to data analysis and inference in complex systems (8), and has become a popular method of machine learning. Based on Bayes' theorem, BN can effectively perform most data mining tasks, such as prediction, attribution, and classification (9), and has been applied in prognostic prediction, treatment decision-making, and other fields. For instance, Bradley et al. constructed a BN model for prognostic prediction of patients with pancreatic ductal adenocarcinoma using inflammatory markers, tumor factors, tumor markers, patient factors, response to neoadjuvant treatment, tumor pathology, and postoperative chemoradiotherapy, and its area under the curve (AUC) reached 80% (10). Nandra et al. constructed a 1-year survival prediction BN model of patients with bone sarcoma based on five variables (age, tumor size, tumor grade, metastasis, and pathologic fracture) with an AUC of 76.7%, and the conditional relationship among these variables was also found (11). Cong L et al. confirmed that patients with advanced gallbladder adenocarcinoma can obtain a better prognosis by R0 resection through the BN model, which would be helpful for clinical decision-making of gallbladder adenocarcinoma treatment (12). However, up to date, BN has not been used to predict the prognosis of rectal cancer.

Given this situation, the present study aimed to explore the prognostic factors based on the clinical parameters of rectal cancer patients, construct a prognostic prediction model using BN, and compare the prediction efficacy of BN with a nomogram.

## Patients and Methods

### Patients

This study was performed in the Department of Digestive Surgery, Xijing Hospital. A total of 705 patients with rectal cancer were enrolled from January 2015 to December 2017 and were followed up by telephone every half year till March 2021. Patients who met the following criteria were included in the study: (1) being diagnosed with adenocarcinoma; (2) radical resection was performed. The exclusion criteria were as follows: (1) having a history of malignant tumors; (2) having other malignant tumors; (3) having distant metastasis; (4) having adjacent organ invasion; (5) having preoperative radiotherapy; and (6) having been lost to follow-up within 36 months. The study followed the Declaration of Helsinki, and the ethical application was approved by the medical ethics committee of Xijing hospital (ethical code: KY20212146-F-1).

Included clinicopathological factors were as follows: age, gender, BMI, ABO blood type, preoperative serum level of CEA, CA19-9, and CA125, preoperative chemotherapy, surgical type, operation time, tumor size, macropathology type, lymphovascular invasion, tumor differentiation status, KRAS mutation, T stage, N stage, postoperative radiotherapy, and postoperative chemotherapy. The cut-off values of BMI were 18.5 and 25 Kg/m^2^, which were the criteria for classifying low weight, normal weight, and overweight. The cut-off values of CEA, CA19-9, and CA125 levels were their normal and abnormal criteria (5 ng/ml, 37 U/ml, and 35 U/ml) respectively. The optimal cut-off values of age, operation time, and tumor size were calculated using X-Tile software (Yale University, V3.6.1) based on the training dataset.

### Verification of Consistency Between Training and Testing Datasets

The dataset was randomly divided into a training dataset (70%, *n* = 493) and a testing dataset (30%, *n* = 212) using the “rand” function in Microsoft Excel. The distribution differences of variables between the training and testing datasets were analyzed using Fisher's exact test through GraphPad Prism 8.

### Variables' Selection

Overall survival (OS) analysis for the entire cohort was calculated by the Kaplan–Meier method through GraphPad Prism 8 (GraphPad Software, Inc., USA). A univariate Cox regression analysis was performed by SPSS software (version 25, SPSS Inc., USA) based on the training dataset. Variables with *p* < 0.05 were considered significant and used to construct the BN model.

### Construction of the BN Model

The BN model represents variables as nodes, and the connections between nodes as directed edges from the parent node to the child node (9). Since BN can only analyze discrete data, all continuous variables are converted to discrete variables. OS was divided into two categories: dead within 36 months or survived more than 36 months. The Tree Augmented Naïve (TAN) Bayes method was used for the BN model construction based on the training dataset through BayesiaLab software (Bayesian Ltd. Co., France). The TAN algorithm includes four steps: compute the mutual information function among the different variables included; build an undirected graph; build a maximum weighted spanning tree; and convert the undirected tree to a directed one by choosing the root variable and setting the direction of the edges to outward from it (13, 14), which was autonomously calculated by BayesiaLab software.

### Construction of the Nomogram

Independent variables were screened through Cox proportional risk regression using the training dataset. The variables with statistical significance in the univariate analysis were included in the multivariate Cox regression survival analysis. Variables with *p* < 0.05 were considered as independent variables and applied to construct the Cox regression-based nomogram through R software (www.r-project.org, version 4.0.5). The concordance index (C-index) and calibration curve were calculated or produced using R software to reflect the discrimination of the nomogram.

### Model Validation and Assessment

The testing dataset was used for model validation and assessment through the receiver operating characteristic (ROC) curve, which was constructed using R software, and the area under the curve (AUC) was computed to assess the performance of the two models.

## Results

### General Characteristics of the Study Population

**Figure 1:**
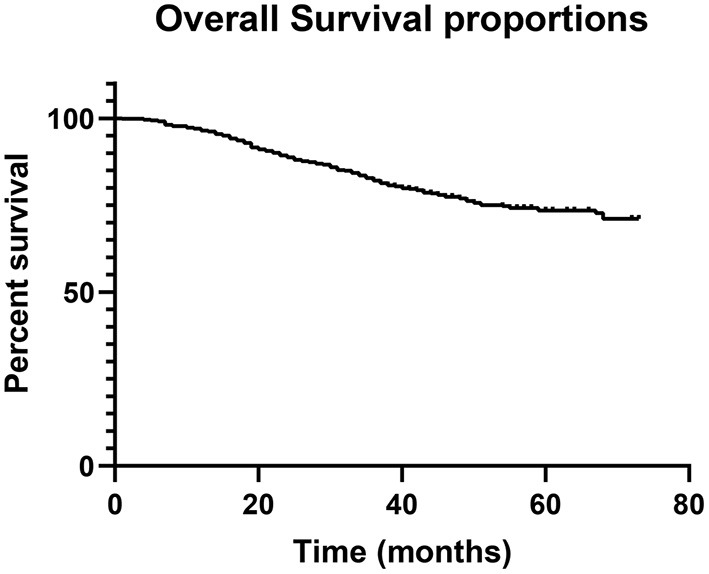
Overall survival of the entire cohort.

There were 425 male patients and 280 female patients. The median age was 60 years (21–87). During follow-up, 170 patients died, accounting for 24.1% of the entire population. The 1-, 3-, and 5-year OS rate was 96.5, 82.1, and 73.5%, respectively (Figure 1). The study cohort was randomly divided into a training dataset (493 cases, 70%) and a testing dataset (212 cases, 30%). The optimal cut-off value was 73 years for age, 205 min for operation time, and 3.5 cm for tumor size, respectively. All the parameters were comparable between the two datasets (Table 1). The characteristics of the entire cohort are summarized in Table 1.

**Table 1:** Clinicopathological characteristics of the entire cohort and the comparison of variable consistency between training and testing dataset.

**Characteristics**	**Value**	***n*** **(%)**	**Training**	**Testing**	***P*** **-value**
**Age**					0.794
≤ 73 y	0	627 (88.9%)	437	190	
>73 y	1	78 (11.1%)	56	22	
**Gender**					0.801
Male	0	425 (60.3%)	299	126	
Female	1	280 (39.7%)	194	86	
**BMI**					0.529
Low (BMI <18.5 Kg/m2)	1	49 (7.0%)	31	18	
Normal (18.5 Kg/m2 ≤ BMI <25 Kg/m2)	2	473 (67.0%)	331	142	
High (BMI ≥25 Kg/m2)	3	183 (26.0%)	131	52	
**ABO blood type**					0.310
A	1	187 (26.5%)	121	66	
B	2	243 (34.5%)	172	71	
O	3	210 (29.8%)	152	58	
AB	4	65 (9.2%)	48	17	
**CEA**					0.792
≤ 5 ng/mL	0	480 (68.1%)	334	146	
>5 ng/mL	1	225 (31.9%)	159	66	
**CA19-9**					0.562
≤ 37 U/mL	0	643 (91.2%)	452	191	
>37 U/mL	1	62 (8.8%)	41	21	
**CA125**					0.664
≤ 35 U/mL	0	679 (96.3%)	476	203	
>35 U/mL	1	26 (3.7%)	17	9	
**Preoperative chemotherapy**					0.880
Without chemotherapy	0	648 (91.9%)	452	196	
With chemotherapy	1	57 (8.1%)	41	16	
**Surgical type**					0.999
Open	0	160 (22.7%)	112	48	
Laparoscopic	1	545 (77.3%)	381	164	
**Operation time**					0.999
≤ 205 min	0	590 (83.7%)	413	178	
>205 min	1	115 (16.3%)	80	34	
**Macropathology type**					0.938
Protuberance	1	125 (17.7%)	87	38	
Ulcer	2	516 (73.2%)	360	156	
Others	3	64 (9.1%)	46	18	
**Tumor size**					0.499
≤ 3.5 cm	0	266 (37.7%)	182	84	
>3.5 cm	1	439 (62.3%)	311	128	
**Differentiation status**					0.232
Well	1	231 (32.8%)	166	65	
Morderate	2	413 (58.6%)	290	123	
Poor	3	61 (8.7%)	37	24	
***T*** **stage**					0.166
Tis/1	1	66 (9.4%)	42	24	
T2	2	168 (23.8%)	116	52	
T3	3	447 (63.4%)	322	125	
T4	4	24 (3.4%)	13	11	
***N*** **stage**					0.315
N0	0	398 (56.5%)	278	120	
N1	1	206 (29.2%)	150	56	
N2	2	101 (14.3%)	65	36	
**Lymphovascular invasion**					0.248
No invasion	0	488 (69.2%)	348	140	
Invasion	1	217 (30.8%)	145	72	
**KRAS mutation**					0.174
Wild type	0	540 (76.6%)	385	155	
Mutant	1	165 (23.4%)	108	57	
**Postoperative radiotherapy**					0.679
Without radiotherapy	0	567 (80.4%)	394	173	
With radiotherapy	1	138 (19.6%)	99	39	
**Postoperative chemotherapy**					0.660
Without chemotherapy	0	224 (31.8%)	154	70	
With chemotherapy	1	481 (68.2%)	339	142	

### Univariate Analysis

The prognostic predictors for the training dataset were analyzed using univariate Cox regression analysis (Table 2). The results showed that age, CEA, CA19-9, CA125, preoperative chemotherapy, macropathology type, tumor size, differentiation status, T stage, N stage, lymphovascular invasion, KRAS mutation, and postoperative chemotherapy were associated with the prognosis of patients with rectal cancer.

**Table 2:** A univariate Cox regression analysis for overall survival (OS) of the training dataset.

**Variable**	**HR**	**95% CI**	***P*** **-value**
Age	1.482	1.048–2.152	0.045
Gender	0.799	0.540–1.184	0.264
BMI	0.988	0.697–1.401	0.948
Blood type	0.996	0.814–1.217	0.965
CEA	2.028	1.394–2.951	<0.001
CA19-9	2.838	1.730–4.656	<0.001
CA125	3.054	1.486–6.276	0.002
Preoperative chemotherapy	2.243	1.336–3.763	0.002
Surgical type	1.031	0.660–1.611	0.894
Operation time	1.651	0.993–2.563	0.052
Macropathology type	1.335	1.022–1.635	0.041
Tumor size	1.689	1.110–2.570	0.014
Differentiation status	1.920	1.400–2.633	<0.001
*T* stage	2.268	1.603–3.209	<0.001
*N* stage	1.765	1.389–2.242	<0.001
Lymphovascular invasion	1.762	1.202–2.582	0.004
KRAS mutation	1.569	1.039–2.369	0.032
Postoperative radiotherapy	0.963	0.599–1.549	0.877
Postoperative chemotherapy	0.506	0.347–0.738	<0.001

### BN Model Development

**Figure 2:**
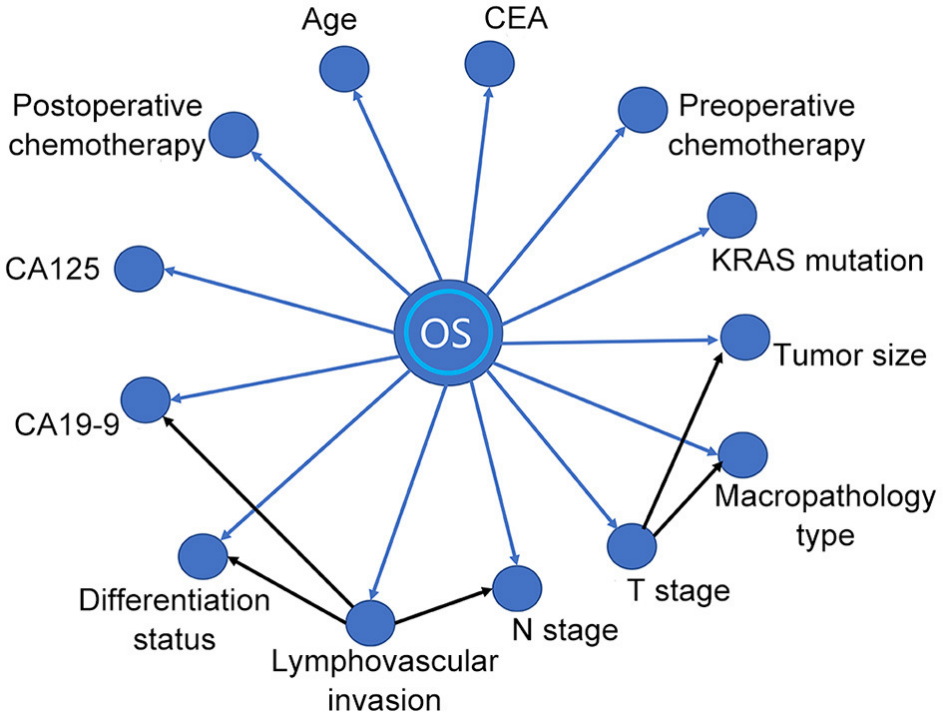
A Bayesian network (BN) model for prognostic factors of the training dataset.

A BN model based on the training dataset was established using the above-mentioned prognostic predictors. The model included the relationship between OS and prognostic factors, as well as the correlation among the factors (Figure 2). As shown in the BN model, OS was affected by 13 variables. In addition, the model also identified cause-and-effect associations between the T stage and other two variables (tumor size and macropathology type), lymphovascular invasion, and other three variables (CA19-9, N stage, and differentiation status). That means tumor size and macropathology type were conditionally associated with T stage, and CA19-9, N stage, and differentiation status were conditionally associated with lymphovascular invasion.

### Nomogram Development

**Figure 3:**
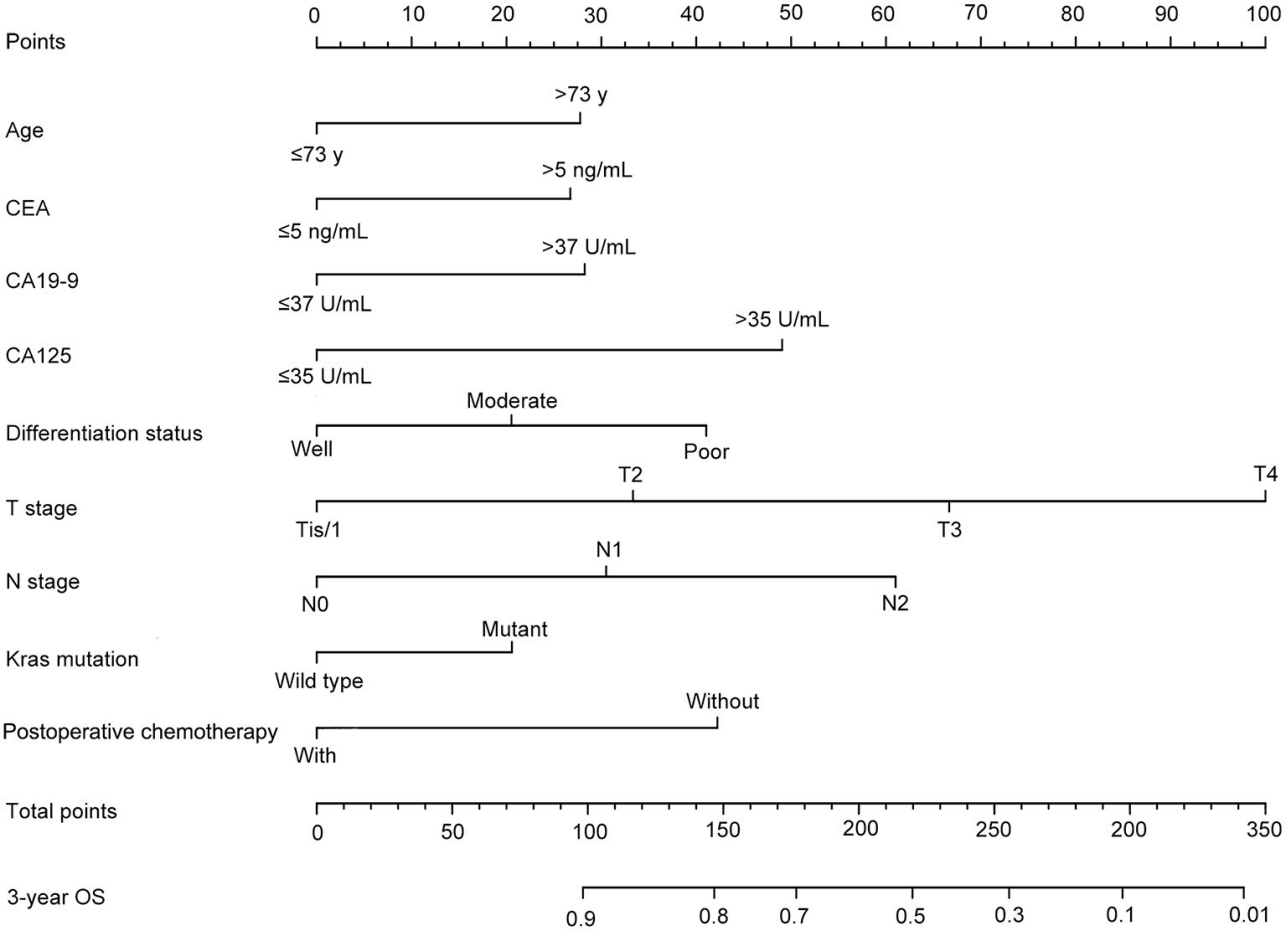
A nomogram for predicting the 3-year overall survival (OS) of the training dataset.

**Figure 4:**
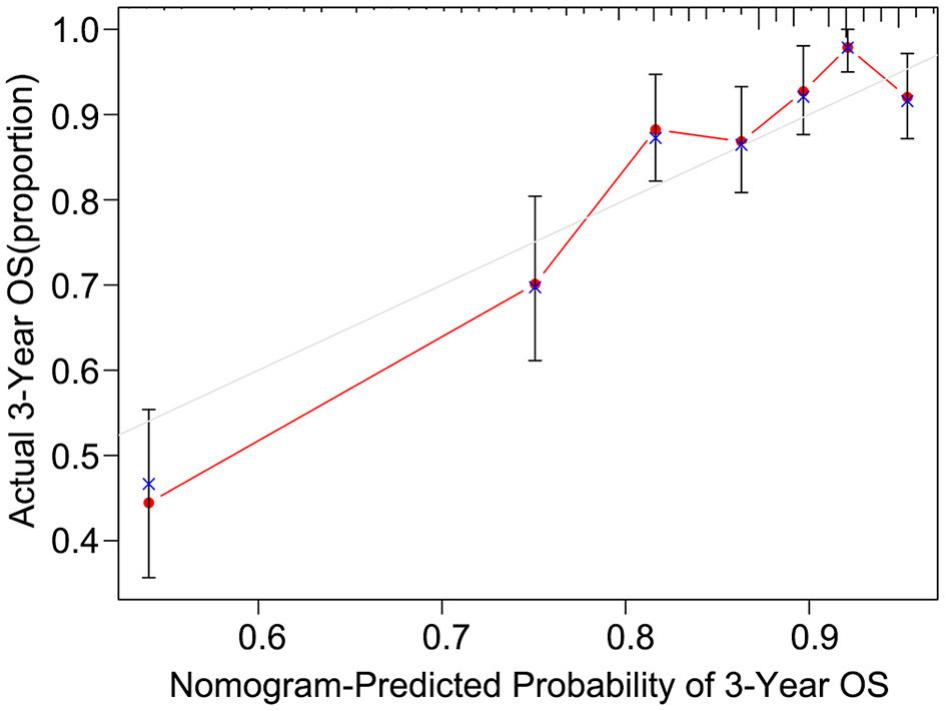
The calibration curve of the nomogram for predicting the 3-year OS of the training dataset.

After univariate analysis, multivariate Cox regression analysis was performed to determine which variables were independent prognostic factors. The results showed that age, preoperative serum CEA, CA19-9, and CA125, differentiation status, T stage, N stage, KRAS mutation, and postoperative chemotherapy were independent prognostic factors for the prognosis of rectal cancer (Table 3). Then, the nomogram was constructed based on the 9 independent prognostic variables (Figure 3). The C-index of the nomogram was 0.745. The calibration curve is shown in Figure 4.

**Table 3:** A multivariate Cox regression analysis for OS of the training dataset.

**Variable**	**HR**	**95% CI**	***P*** **-value**
Age	1.588	1.033–2.440	0.035
CEA	1.499	1.075–2.091	0.017
CA19-9	1.613	1.032–2.473	0.039
CA125	2.074	1.136–3.786	0.018
Preoperative chemotherapy	1.623	0.985–2.074	0.061
Macropathology type	1.111	0.873–1.413	0.392
Tumor size	1.273	0.905–1.789	0.165
Differentiation status	1.382	1.069–1.787	0.014
*T* stage	1.602	1.200–2.139	0.001
*N* stage	1.521	1.209–1.914	<0.001
Lymphovascular invasion	1.152	0.813–1.632	0.427
KRAS mutation	1.469	1.041–2.073	0.029
Postoperative chemotherapy	0.531	0.389–0.726	<0.001

### Assessment of Model Efficacy

**Figure 5:**
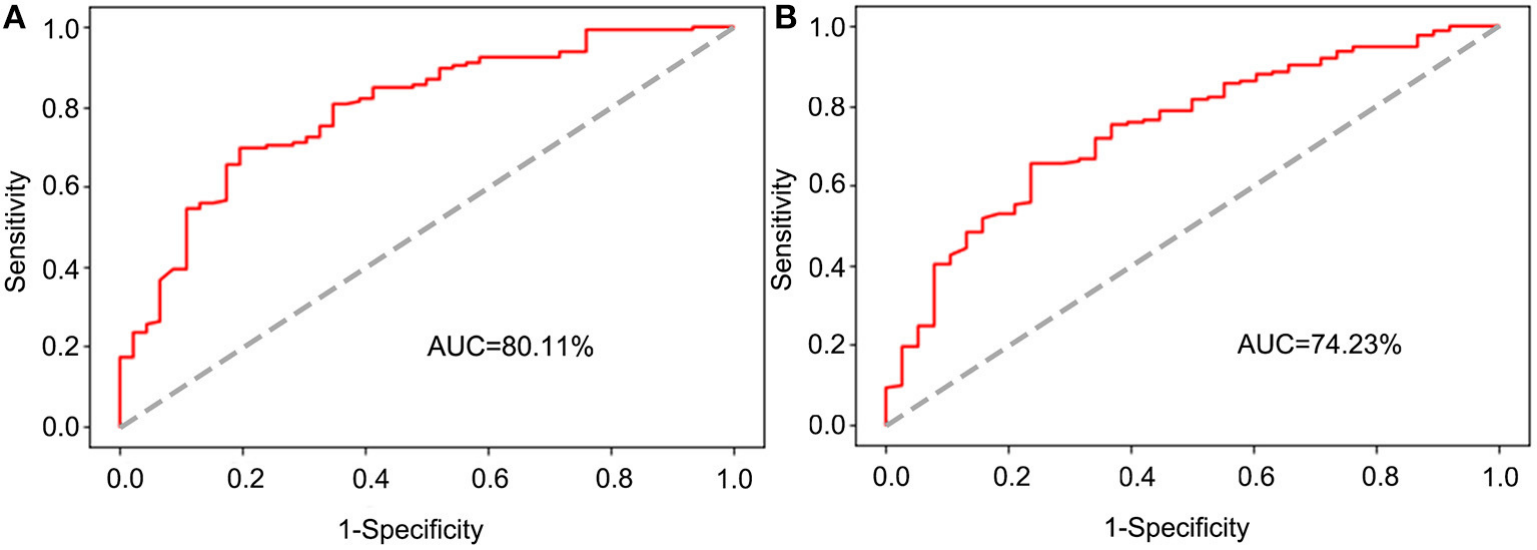
The receiver operating characteristic (ROC) curve for validation of the BN and nomogram model based on the testing dataset. **(A)** The ROC curve for validation of the BN model. **(B)** The ROC curve for validation of the nomogram.

To explore whether the BN model is better than the nomogram, the testing dataset was used to assess the performance of the BN model and the nomogram. The ROC curves of the two models were established, respectively (Figure 5), and the AUC for the BN model was higher than that for the nomogram (80.11 vs. 74.23%).

## Discussion

Rectal cancer is one of the most concerned cancer types in the world with high morbidity and mortality (1). Surgical resection remains the primary treatment for rectal cancer (15). The establishment of a prognostic prediction model for postoperative patients will help medical workers and patients to evaluate the prognostic status and to make decisions on examination and treatment programs. A nomogram has been widely used for cancer prediction, which plays a role in personalized prediction for patients with rectal cancer (6, 7). However, it ignores the interaction of prognostic factors and their joint effect on cancer prognosis. Thus, a new modeling method is required to compensate for this deficiency. With the rapid development of machine learning algorithms, researchers propose that they can be used to supplement traditional statistical methods in the field of medical research. The BN model is a common and effective method in the field of machine learning, which can mine unknown information from observed data, and plays an important role in clinical decision-making, prognostic research, and other fields (12, 16). However, BN has not been used to predict the prognosis of rectal cancer so far. In this study, we constructed a BN model for prognostic prediction of rectal cancer based on the clinicopathological characteristics of patients using the Bayesian network for the first time and demonstrated that the BN model performed better than the nomogram.

In recent years, there have been some studies focusing on the prognosis prediction of patients with rectal cancer. Zhao et al. established a nomogram for the prognosis prediction of metastatic rectal cancer by using the patients' data from the U.S. National Cancer Database (17). Song et al. studied the prognostic factors of patients with locally advanced rectal cancer receiving neoadjuvant chemoradiotherapy and established a nomogram for prognostic prediction (18). In addition, another study explored the prognostic predictive role of pathologic features in locally advanced rectal cancer using a nomogram (7). Liu et al. established a prognostic prediction nomogram for middle-aged and older patients with rectal cancer using data from Surveillance, Epidemiology, and End Results database (19). So far, the prognostic prediction models for patients with rectal cancer were almost based on nomograms, which neglected the relationships among prognostic factors and their mutual influences.

Nowadays, using machine learning tools, such as BN, to build prognostic prediction models is becoming more and more widespread (10, 13, 20). Based on the combination of graph theory and probability theory, BN can reduce the complexity of reasoning (21). It is noteworthy that the BN model has been used to predict the prognosis of some kinds of malignant tumors, such as gallbladder cancer (9), pancreatic ductal adenocarcinoma (10), lung cancer (16), and bone sarcomas (11). So far as we know, although Fielding et al. have used Bayesian theory to predict the prognosis of patients with colon cancer (22), BN has not been used to predict the prognosis of patients with rectal cancer. The BN model could not only predict the prognosis but also identify the correlation between prognostic factors. The connection arrow between variables in the BN model represents the conditional probability from the parent node to the child node, which means, given the state of the parent node, the probability of certain events occurring in the child node would be affected (23). In this study, our BN model found the following conditional dependencies between variables: the state of the T stage would affect the probability of tumor size and macropathology type. Similarly, the state of vascular invasion would affect the probability of preoperative serum CA19-9, N stage, and differentiation status. These influences between variables form a joint probability distribution and make it possible to use the BN model to predict the personalized prognosis even if a few variables are missing, although the efficacy of prediction may be reduced (24). Such findings would also provide a reference direction for further study of the underlying pathological or pathophysiological mechanisms of the associations between variables. These capabilities are what nomograms do not have.

From the perspective of variable types, a nomogram is based on independent prognostic factors, while BN does not require independent variables, but integrates the association and joint effect of prognostic variables. These differences might make the prediction power and accuracy of the BN model better than that of the nomogram. In addition, Wu et al. found that the BN model performed better than the nomogram in prognosis prediction of gallbladder cancer (9), which was similar to our findings. To construct the BN model, continuous variables need to be converted into categorical variables, while the independent variables of the nomogram can be continuous, which makes the data processing more complicated before the BN model construction. In general, BN is easier to operate than the nomogram and more convenient for clinicians because it eliminates the process of analyzing independent prognostic factors and only requires BayesiaLab software.

During the follow-up, all patients were followed for more than 36 months except those who died within 36 months. This allows the study to predict whether patients will survive beyond 36 months after resection of rectal cancer with a sufficient follow-up period. Since the BN model can only predict categorical variables, patients' survival time needs to be dichotomized. To predict the probability of survival over 1-, 3-, or 5-year, it would be necessary to dichotomize the survival time of patients correspondingly and construct the corresponding BN prediction models accordingly (25), while a nomogram would only need to construct a model for one time to predict the survival probability of patients with different survival periods, which is a disadvantage of BN over nomogram. Since BN can only analyze survival time but not survival status in the process of modeling, premature loss to follow-up may affect machine learning, which is also a disadvantage compared with the nomogram and may affect the prediction efficiency of the model.

There are some limitations to the present study. First, this study was a single-center retrospective study. Second, although the follow-up time of patients in our study was up to 74 months, most patients were followed up for <5 years, thus it would not be possible to construct a prognostic model of 5-year OS. Third, some risk factors reported in other studies that may affect the prognosis of rectal cancer, such as perineural invasion (26), microsatellite stability status (27), and other gene mutation status (28, 29), were not included in this study due to lack of relevant testing or data availability. In the future, we may carry out multi-center studies with more cases, longer follow-ups, and more parameters, to construct a more accurate BN model for prognostic prediction of patients with rectal cancer.

In conclusion, this study analyzed clinicopathological factors influencing the prognosis of patients with rectal cancer after radical resection, constructed a 3-year OS prediction BN model for the first time, and investigated the underlying cause-and-effect relationships among variables. We also demonstrated that the BN model performed better than the nomogram. The BN model constructed in this study can be used for a personalized evaluation of the prognosis of patients with rectal cancer and provide clinicians with an accurate prognostic evaluation tool.

## Data Availability Statement

The raw data supporting the conclusions of this article will be made available by the authors, without undue reservation.

## Ethics Statement

The studies involving human participants were reviewed and approved by the Medical Ethics Committee of Xijing Hospital. Xijing hospital, Fourth Military Medical University, Xi'an, China. The patients/participants provided their written informed consent to participate in this study.

## Author Contributions

RL, FF, and JZ designed the study. RD, GZ, HW, GR, and XD collected the data. RL, CZ, KD, HD, and ZX analyzed the data. RL, CZ, ZC, and LD visualized the data. RL drafted the manuscript. FF and JZ revised the manuscript. All authors have read and approved the final manuscript. All authors contributed to the article and approved the submitted version.

## Funding

This study was supported by the National Natural Science Foundation of China (Grant No. 82072655).

## Conflict of Interest

The authors declare that the research was conducted in the absence of any commercial or financial relationships that could be construed as a potential conflict of interest.

## Publisher's Note

All claims expressed in this article are solely those of the authors and do not necessarily represent those of their affiliated organizations, or those of the publisher, the editors and the reviewers. Any product that may be evaluated in this article, or claim that may be made by its manufacturer, is not guaranteed or endorsed by the publisher.

## Abbreviations

AUC: area under the curve
BMI: body mass index
BN: Bayesian network
C-index: concordance index
OS: overall survival
ROC: receiver operating characteristic
TAN: Tree Augmented Naïve.
